# Tracing Blue Carbon Flows Across Diverse Seascapes

**DOI:** 10.1111/gcb.70420

**Published:** 2025-08-15

**Authors:** Christopher J. Fulton, Diego R. Barneche, Kay Davis, Cal Faubel, Cecilia Pascelli, Julie Vercelloni, Shaun K. Wilson

**Affiliations:** ^1^ Australian Institute of Marine Science Crawley, Western Australia Australia; ^2^ Oceans Institute University of Western Australia Crawley, Western Australia Australia; ^3^ Australian Institute of Marine Science Cape Cleveland Queensland Australia; ^4^ Centre for Data Science Queensland University of Technology Brisbane Queensland Australia

**Keywords:** eDNA, lipids, macroalgae, mixing models, plankton, SPOM, stable isotopes

## Abstract

Plants occupying coastal ecosystems draw in carbon dioxide (CO_2_) from the air and water around them during photosynthesis. A fraction of this CO_2_ becomes fixed into plant biomass and can eventually contribute to the blue carbon pool—organic carbon (C_org_) sequestered in slow‐turnover sinks. An important step in protecting and enhancing this natural carbon sequestration pathway is determining the relative contributions of different coastal plants to this blue carbon pool in durable sinks. We compiled a global dataset of coastal soil carbon measurements and used a Bayesian hierarchical meta‐regression model to explore the relative contribution of local (autochthonous) versus external (allochthonous) sources of C_org_ in the soils beneath tidal saltmarsh, mangrove, and seagrass wetlands. We found most soil C_org_ in coastal wetlands came from allochthonous sources, rather than the habitat‐forming plants. Managing climate‐resilient blue carbon seascapes, therefore, requires an awareness of this portfolio of contributors to soil carbon sequestration. However, study design aspects such as soil sampling depth, levels of sample replication, and the modeling approach used to trace C_org_ can have pronounced effects on the estimated contributions of different carbon sources. We outline a roadmap for improving how we track the various drivers of soil carbon sequestration in diverse mosaics of coastal vegetation.

## Graphical abstract

Plants occupying coastal wetlands draw in carbon dioxide from the air and water to grow plant tissues via photosynthesis, some of which is buried under the soil where it is sequestered from the atmosphere. We collated a global dataset of soil carbon measurements to explore the relative contribution of local (autochthonous) versus external (allochthonous) sources of soil organic carbon beneath saltmarsh, mangrove, and seagrass wetlands. We found that most soil carbon in these wetlands came from a diversity of allochthonous sources, which highlights the need for holistic seascape management to protect this portfolio of contributors to soil carbon sequestration.

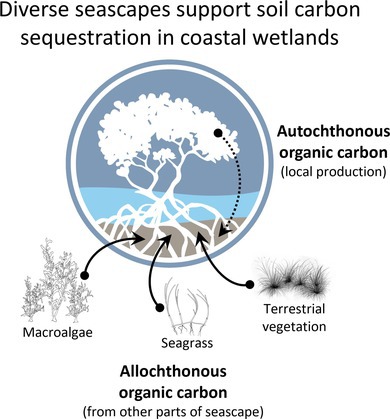

## 1 Introduction

Blue carbon refers to the natural binding and storing of carbon by coastal and oceanic ecosystems (Nellemann et al. 2009). Building upon decades of research (Smith 1981; Lovelock and Duarte 2019; Macreadie et al. 2019; Howard et al. 2023), there has been a global push to protect and restore blue carbon ecosystems to enable this natural pathway of carbon sequestration to reduce the severity of greenhouse gas‐induced climate change (IPCC 2014; Macreadie et al. 2021; IPBC 2023). Much of this attention has been directed to saltmarsh, mangrove forests, and seagrass beds that are listed within international guidelines for national greenhouse gas inventories (IPCC 2014; Howard et al. 2017; Macreadie et al. 2021). Within this momentum for action, there are still considerable scientific uncertainties around the major sources, pathways, and rates of carbon sequestration into coastal wetland soils (Lovelock and Duarte 2019; Macreadie et al. 2019; Howard et al. 2023).

Organic carbon (C_org_) sequestration into wetland soils begins with photosynthetic organisms fixing inorganic carbon into biomass, some of which is subsequently buried in areas where there is active accumulation and stabilization of new soil layers over time (Duarte et al. 2005; Howard et al. 2017; Macreadie et al. 2019). There are two major pathways for the delivery of new C_org_ into soils: (1) autochthonous carbon fixed by the habitat‐forming organism and buried in the soil beneath where they grow (e.g., mangrove organic carbon deposited into mangrove soil), and (2) allochthonous carbon fixed by other photo‐autotrophic sources growing in a connected seascape (Duarte and Cebrian 1996; Hyndes et al. 2013; Santos et al. 2021). Depending on the climate, hydrology, and geomorphic setting, there can be a huge array of allochthonous sources delivered into a given soil area via tidal flows and other water circulation features (Hyndes et al. 2013; Santos et al. 2021; Zhang et al. 2024). Quantifying the extent of these different blue carbon flows is necessary for designing the most effective approaches for coastal management, adaptation, and restoration of connected seascapes resilient to climate change.

Tracing C_org_ contributions into marine soils can be technically challenging when there are many potential sources (often called “end‐members”). Mixing models are typically used to unravel the sources of organic carbon present within a soil sample using chemical tracers (Stock et al. 2018; Geraldi et al. 2019; Macreadie et al. 2019). The most widely used tracers are stable isotopes (Parnell et al. 2013; Stock et al. 2018; Macreadie et al. 2019), such as the ratio (δ) of heavy to light isotopes of organic carbon (13C_org_:12C_org_). The earliest mixing models were based on mass balance equations; while they provided early insights into source contributions to soil C_org_, they were based on one or two tracers, very few potential sources and often ran into issues of overlapping source δ13C_org_ signatures (Phillips and Gregg 2003; Stock et al. 2018; Macreadie et al. 2019). Adoption of additional tracers and Bayesian approaches (e.g., MixSIR, MixSIAR) has since allowed many potential sources to be considered in mixing models (Parnell et al. 2013; Neubauer and Jensen 2015; Stock et al. 2018). Any synthesis of estimated blue carbon contributions to soil C_org_ must account for the constraints imposed by these different analytical approaches alongside other sampling and environmental effects.

**FIGURE 1:**
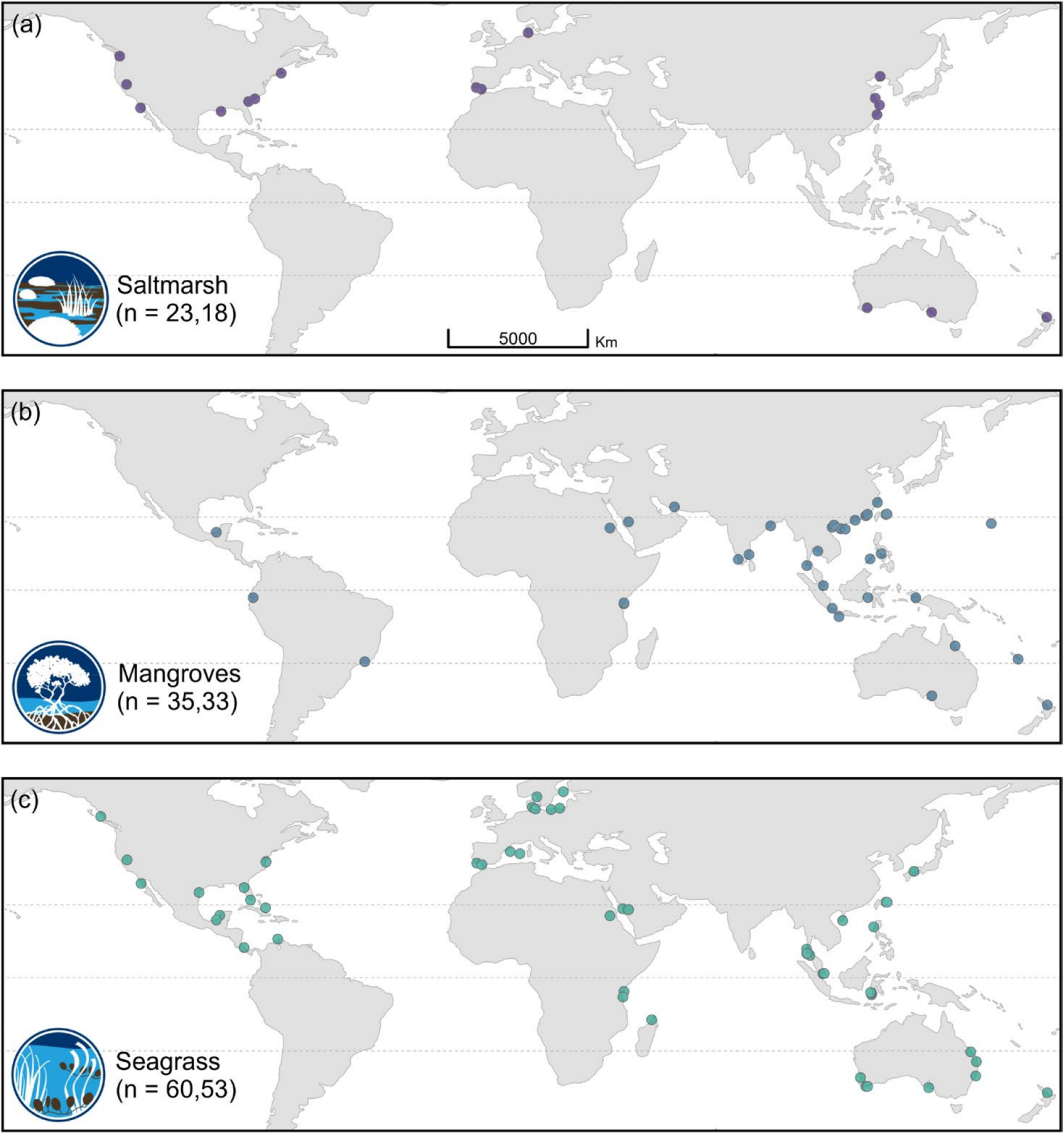
Global distribution of 110 independent estimates of organic carbon sources contributing to the soil carbon mixture in three coastal wetland types: (a) saltmarsh, (b) mangrove, and (c) seagrass. Dotted center lines indicate the equator, with tropics of Cancer and Capricorn above and below. Numbers in parentheses are the number of independent datasets and published studies (respectively) for each habitat. Note that studies often included multiple habitat types, several independent estimates per habitat type (using different tracer families), and/or estimates from widely spaced locations (different countries) per habitat type.

Empirical estimates for the relative contribution of different plants to organic carbon sequestration in coastal soils have been accumulating for many decades. We used a meta‐analysis of 78 such studies to explore which photoautotrophs may be important contributors to soil organic carbon sequestration across a global spread of tropical and temperate wetlands (Figure 1). We applied a Bayesian meta‐regression model to the ratio of autochthonous to allochthonous sources of soil organic carbon in three vegetated tidal wetlands (saltmarsh, seagrass, mangrove) to explore the effect of different sampling and analytical approaches. In doing so, we offer a roadmap of recommendations and solutions to address the limitations of current approaches and advance progress toward understanding and managing our diverse blue carbon seascapes.

## 2 Materials and Methods

### 2.1 Data Search, Extraction, and Collation

Peer‐reviewed published studies of potential sources of soil organic carbon in coastal and marine settings were identified within the Scopus database (www.scopus.com, Elsevier B.V., publication date range 1971–2023) using the following combination of search terms and Boolean operators to scan the Title, Abstract and Keywords fields: sediment* AND carbon AND organic AND source* OR contribution* OR provenance* OR tracer* OR eDNA OR isotope* AND blue OR seagrass* OR mang* OR marsh* OR tidal AND wetland* AND NOT beach* OR seep* OR ore* OR metal* OR hydrocarbon* OR pollution. This search was first performed on July 26, 2023 to yield 1138 potential articles, and a rerun on February 6, 2024 captured an additional 14 articles published up to the end of 2023. These 1152 potential articles were manually screened to a subset of articles that, as a minimum, clearly reported all of the following: unique empirical data on organic carbon content in marine soils within tidal/subtidal regions, soil sampling depth(s), spatial sampling location(s), sampled wetland type and quantitative data to trace the autotrophic sources of soil C_org_.

The literature search and screening process yielded a total of 116 studies published in peer‐reviewed journals (see list in Data Sources), from which 110 habitat‐specific independent estimates of soil organic carbon were obtained from 78 peer‐reviewed published studies spanning publication dates from 1977 to 2023 (see data online https://doi.org/10.25845/jt3q‐4y65); note that 24 studies sampled two or more habitat types and/or used two or more tracers within mixing models for the one study. Data were unable to be used/extracted from the other studies due to: (a) being syntheses entirely based on data from other studies; (b) unclear sampling designs that did not adequately link reported data to specific wetland types, locations and/or levels of replication; (c) lacking a quantitative basis for tracing specific sources into soil C_org_ mixtures (e.g., mixing model) or the data on specific C_org_ sources needed to perform our own mixing model calculations (e.g., stable isotope signatures clearly linked to each replicate sediment mixture and clear carbon sources in the seascape); (d) being conducted in polar latitudes (*n* = 2 studies); or (e) failing to report an autochthonous C_org_ contribution (typical for unvegetated mudflats). While many studies claimed to explore soil C_org_ sources, these were broad qualitative comparisons among various sources and sediment mixtures and were excluded according to (c) above.

For each independent estimate, we collated data on a minimum of seven features: (1) wetland type where sediment samples were collected (mangrove, saltmarsh, seagrass), (2) whether the field samples were collected in a tropical (between 23.43613° S and 23.43613° N) or temperate (between 23.43613° and 66.5° North and South of the Equator) climatic region, (3) level of spatial replication underlying mean values (from the Methods sections, site maps, or stated latitude–longitude coordinates), (4) soil depth at which samples were taken, (5) the type of biomarkers measured and applied in the mixing models (δ13C_org_, δ15N, C_org_:N and C_org_:P ratios, eDNA, lipids, lignins), (6) the analytical approach used to predict different C_org_ source contributions to sediment mixtures (standard mixing models, Bayesian mixing models, other), and (7) the reported outcomes of mixing model analyses for specific sources of C_org_. We verified that analyses were based on the measurements of %C_org_ from acidified soil samples. In most cases, we extracted mixing model results directly from the published articles, Supporting Information, and/or linked datasets available online. Four studies did not conduct mixing model analyses, so we extracted the necessary stable isotope measurements to estimate the probable proportions of autochthonous and/or allochthonous sources of C_org_ into the sediment mixture(s) using IsoError (v1.04; Phillips and Gregg 2001; Phillips 2012) software.

### 2.2 Data Structure

All data were collated as spatially discrete soil samples taken within the same wetland type per study, with means and variance terms taken at the highest level of horizontal spatial separation across the seascape (typically within and among sites). Climate (tropical or temperate) was unbalanced across wetlands, with 0%, 83%, and 43% of datasets from tropical locations for saltmarsh, mangrove, and seagrass, respectively. Three primary methods of sediment collection were adopted: coring (84%), surface scrape/grab (9%), and sediment traps (7%); a similar distribution of these collection methods was evident across climates and habitats. Soil sampling depths ranged from zero (surface scrape) down to 3 m (coring), with a mean (± standard deviation) midpoint of 0.20 ± 0.27 m that was broadly similar across habitat types and climates. The most common tracers used to estimate the contribution of different C_org_ sources to the soil mixture were δ13C_org_ (applied in 95% of datasets), δ15N (44%), and C:N ratios (19%). Application of eDNA, lipids, and/or lignin was rare (3%) and typically made outside of a mixing model analysis. In those datasets where stable isotopes were utilized as tracers, 33% were based solely on δ13C_org_, 36% combined δ13C_org_ and δ15N, and 12% used δ13C_org_ with the C:N ratio. Application of those tracers largely involved standard mass balance and linear mixing models (e.g., Schultz and Calder 1976; Phillips and Gregg 2001), with more recent works increasingly using Bayesian mixing models (e.g., SimmR, SIAR, and MixSIAR; Stock et al. 2018) in studies of all three wetland types. Less common were other approaches that included simple ratios of lipid tracers (e.g., taraxerol:total lipids), geometric models (e.g., ternary mixing diagrams; Dittmar et al. 2001), and multivariate analyses of eDNA relative abundance. Accordingly, there was a wide range of end‐members (number of sources) considered in each study, spanning single‐tracer mixing models restricted to two sources, ternary mixing diagrams with three sources, up to 10 end‐members in Bayesian mixing models using multiple tracers, and up to 50 OTUs in eDNA‐based estimates of source contributions.

### 2.3 Meta‐Regression

We explored a range of study design effects on the estimated ratio of autochthonous (auto) to allochthonous (allo) C_org_ contributions into saltmarsh, mangrove, and seagrass wetland soils. We chose this approach because we could identify the autochthonous C_org_ source relevant to the particular wetland vegetation (i.e., saltmarsh plants, mangrove trees and seagrasses) and collate these into a single normally distributed variable (natural log auto:allo, hereafter log‐ratios) that spanned all three wetlands and was amenable to standard multilevel meta‐regression. Calculation of the log‐ratios first required conversion of the different reported measures of variability from each study into standard deviations by assuming normal distributions (hereafter, adjusted SDs; see Supporting Information). We then calculated log‐ratios and their uncertainty using a sampling algorithm that used the reported autochthonous carbon mean and adjusted SDs to sample 1 million values of autochthonous carbon using a 0–100 truncated normal distribution. For each value, we calculated total allochthonous carbon (%) as 100—autochthonous carbon, and then from the 1 million log‐ratios, we calculated a study‐level mean and SD.

A Bayesian multilevel meta‐regression model to quantify the influence of key independent variables on the log‐ratios included wetland type (saltmarsh, seagrass, mangrove), climate (tropical, temperate) and modelling approach used to estimate autochthonous C_org_ contributions into the soil mixture (standard mixing models, Bayesian, and other methods) as independent categorical variables. Number of end‐members (i.e., carbon sources) attempted in the original mixing analyses, sediment sampling depth midpoint (due to the general decay of C_org_ with soil depth; Krüger et al. 2024), and study‐level sampling effort (*n*, range 2–35, median = 6) were included as continuous independent variables. These variables were the most consistently reported across all studies and provided a solid basis for assessing how sampling design and analytical approaches may influence results.

Mean log‐ratios, ${\hat{\theta }}_{\kappa }$, from each study, κ, were assumed to be normally distributed with a mean value, μ, and a standard deviation which is the square root of the sum of both the study‐level SD, ${\hat{\sigma }}_{\kappa }$, which is known, and a residual standard deviation, σ:

$${\hat{\theta }}_{\kappa }\sim Ν\left(\mu ,\sqrt{{{\hat{\sigma }}^{2}}_{\kappa }+\sigma^{2}}\right)$$

$$\mu =\Delta_{\beta_{0_{\kappa }}}+\beta_{T}X_{\kappa }^{T}$$

$$\Delta_{\beta_{0_{\kappa }}}=\zeta_{\beta_{0_{\kappa }}}\sigma_{\beta_{0_{\kappa }}}$$

$$\beta_{T},\zeta_{\beta_{0_{\kappa }}}\sim Ν\left(0,1\right)$$

$$\sigma_{\beta_{0_{\kappa }}},\sigma \sim Ν\left(0.5,0.1\right)$$

where $\beta_{T}$ is a vector of T population‐level (also known as fixed) effects associated with our independent variables coded in matrix (*n* × T) $X_{\kappa }^{T}$. Continuous variables were centred and scaled around their means and standard deviations, respectively, whereas discrete variables were grounded to a reference level. Therefore, the model intercept (which is part of $\beta_{T}$) should be interpreted as the global population mean at the chosen categorical reference levels and when all continuous predictors are zero. $\Delta_{\beta_{0_{\kappa }}}$ represents study‐specific deviations from the model intercept and were estimated as the product between the standardized study‐level effects, $\zeta_{\beta_{0_{\kappa }}}$, and among‐study standard deviations, $\sigma_{\beta_{0_{\kappa }}}$. Importantly, the study serves as a proxy for other unmeasured variables, such as wetland composition and geographical variability. Therefore, as a first approximation, $\sigma_{\beta_{0_{\kappa }}}$ can be interpreted as an *upper bound* estimate of how the ratio of autochthonous to allochthonous sources might be due to these other variables. Prior distributions were set as weakly informative relative to the input data scale (i.e., log response, scaled predictors) following recommendations from Harrer et al. (2021).

We implemented the Bayesian meta‐regression model using the *brms* package version 2.21.0 (Bürkner 2017) in the R language version 4.3.3 (R Core Team 2024). We constructed posterior distributions from 20,000 draws—derived from four parallel Markov Chain Monte Carlo (MCMC) chains of 10,000 steps each, with the first 5000 discarded as the warm‐up period. Convergence diagnostics of MCMC chains were visually assessed using trace plots of parameters, effective sample size measures and the Gelman‐Rubin statistic, $\hat{R}$ (=1 at convergence; Gelman and Rubin 1992) for each model parameter. The full meta‐regression model was compared against a null, intercept‐only meta‐regression model using leave‐one‐out cross‐validation (Vehtari et al. 2017; see Supporting Information). We validated the full model predictions using posterior predictive checks (Figure S1), diagnosed model residuals using the DHARMa package version 0.4.6 (Hartig 2022; Figure S2), and calculated a Bayesian *R* ^*2*^ statistic (Gelman et al. 2019) for the full model (0.41 with 0.29–0.52 95% Bayesian uncertainty intervals). We then visually inspected density plots of the probable contributions of the most commonly reported end‐members (specific sources) to the soil C_org_ mixture by using a sampling simulation approach similar to the one described for the log‐ratios. The code to calculate log‐ratios, model selection and fitting, and production of the simulated density plots is available online (https://www.github.com/open‐AIMS/bc_metareg).

## 3 Results

**FIGURE 2:**
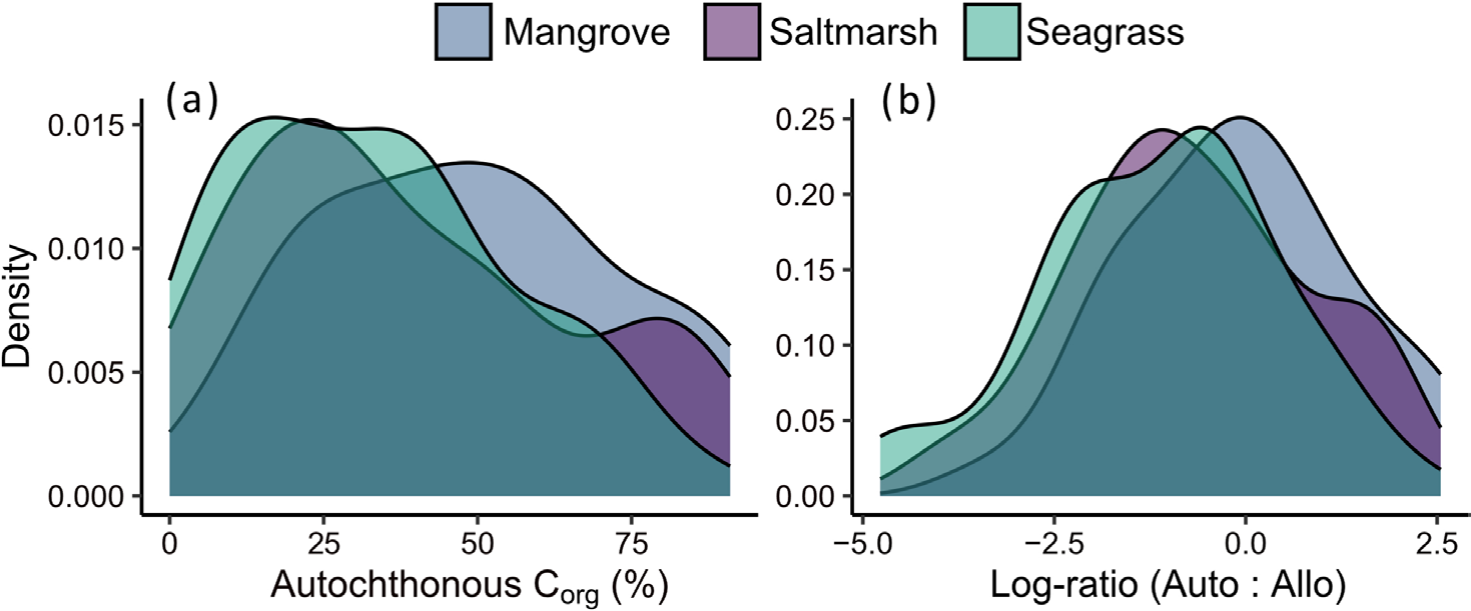
Kernel density representation of (a) the distribution of autochthonous organic carbon (C_org_) sources as reported by independent studies that analyzed soil mixtures from each of saltmarsh (*n* = 20), mangrove (*n* = 34), and seagrass (*n* = 56) wetlands; and (b) the distribution of the log‐ratio of autochthonous to allochthonous C_org_ sources within the three wetland types.

**FIGURE 3:**
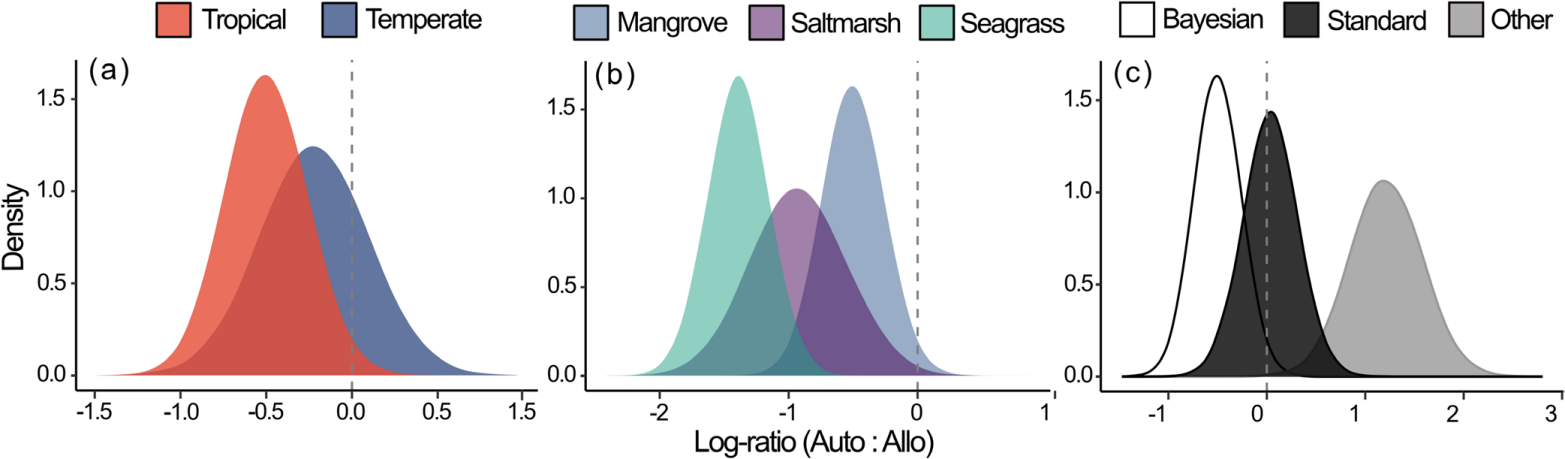
Posterior distribution of effect sizes of categorical model predictors for the log‐ratio of autochthonous to allochthonous organic carbon (C_org_) sources in soils across different (a) climates (mangrove and Bayesian models as reference), (b) habitat types (tropical and Bayesian as reference), and (c) method of source estimation (mangrove and tropical as reference). All effects have been calculated at the median values of all the continuous variables (Figure 4).

**FIGURE 4:**
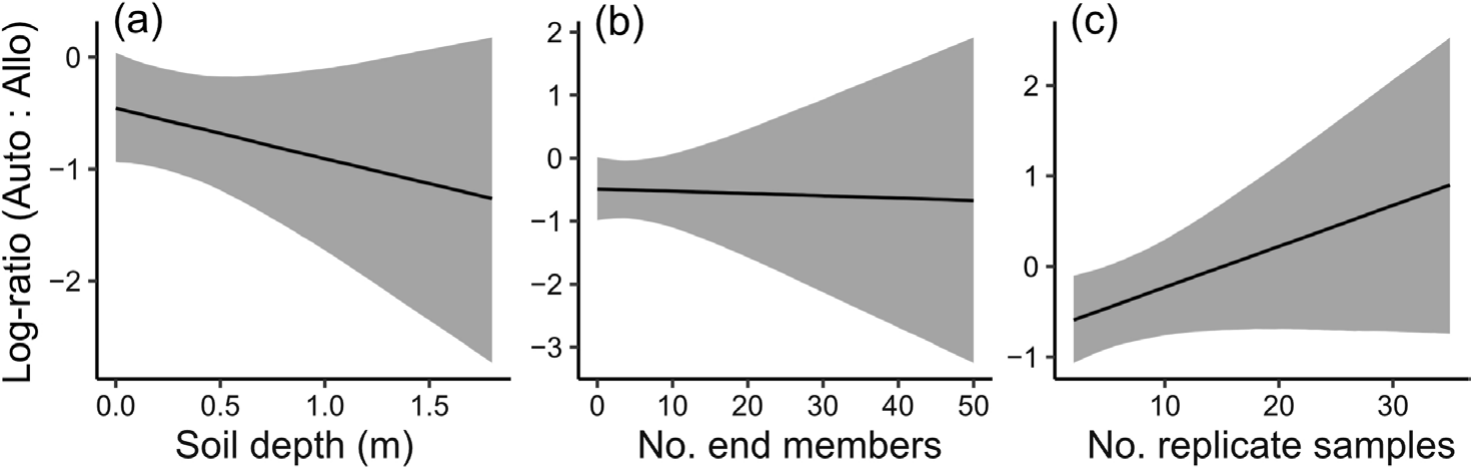
Posterior distributions of continuous model predictors for the log‐ratio of autochthonous to allochthonous organic carbon (C_org_) sources in soils with (a) depth of soil sample, (b) number of end‐member sources considered, and (c) number of replicate soil samples. Continuous covariates have been controlled to their median values. For the purposes of visualization, *x* values have been back converted to the original data scale. Shaded areas are 95% credible intervals.

Variability was high across the independent estimates of autochthonous versus allochthonous contributions to soil C_org_, with a notable tendency toward more allochthonous carbon in all three coastal wetlands (Figure 2). The full meta‐regression model (Table S1) revealed a large between‐study heterogeneity ($\sigma_{\beta_{0_{\kappa }}}$) in log‐ratios (median 0.65 with 0.46–0.81 95% Bayesian uncertainty intervals), which implies an ~3.7‐fold average variation in the ratio of autochthonous to allochthonous carbon sources (i.e., $e^{\left(2\times \sigma_{\beta_{0_{\kappa }}}\right)}$) after accounting for the model fixed‐effect covariates. There was strong evidence for partial habitat effects with more allochthonous C_org_ buried in soils beneath saltmarsh and seagrass wetlands (Figure 3b). Similarly, there was strong evidence Bayesian mixing models estimated higher proportions of allochthonous C_org_ than standard mixing models and other methods (Figure 3c). While there was some evidence for more allochthonous C_org_ contributions into tropical soils (Figure 3a), this should be interpreted with caution due to the unbalanced representation of climates across the three habitat types (Figure 1). Among the continuous predictors, increasing soil sampling depth and number of replicates indicated higher and lower allochthonous C_org_ contributions, respectively, albeit with considerable uncertainty (Figure 4). The number of end‐members had a weak and arguably negligible effect on allochthonous contributions to soil C_org_ (Figure 4).

**FIGURE 5:**
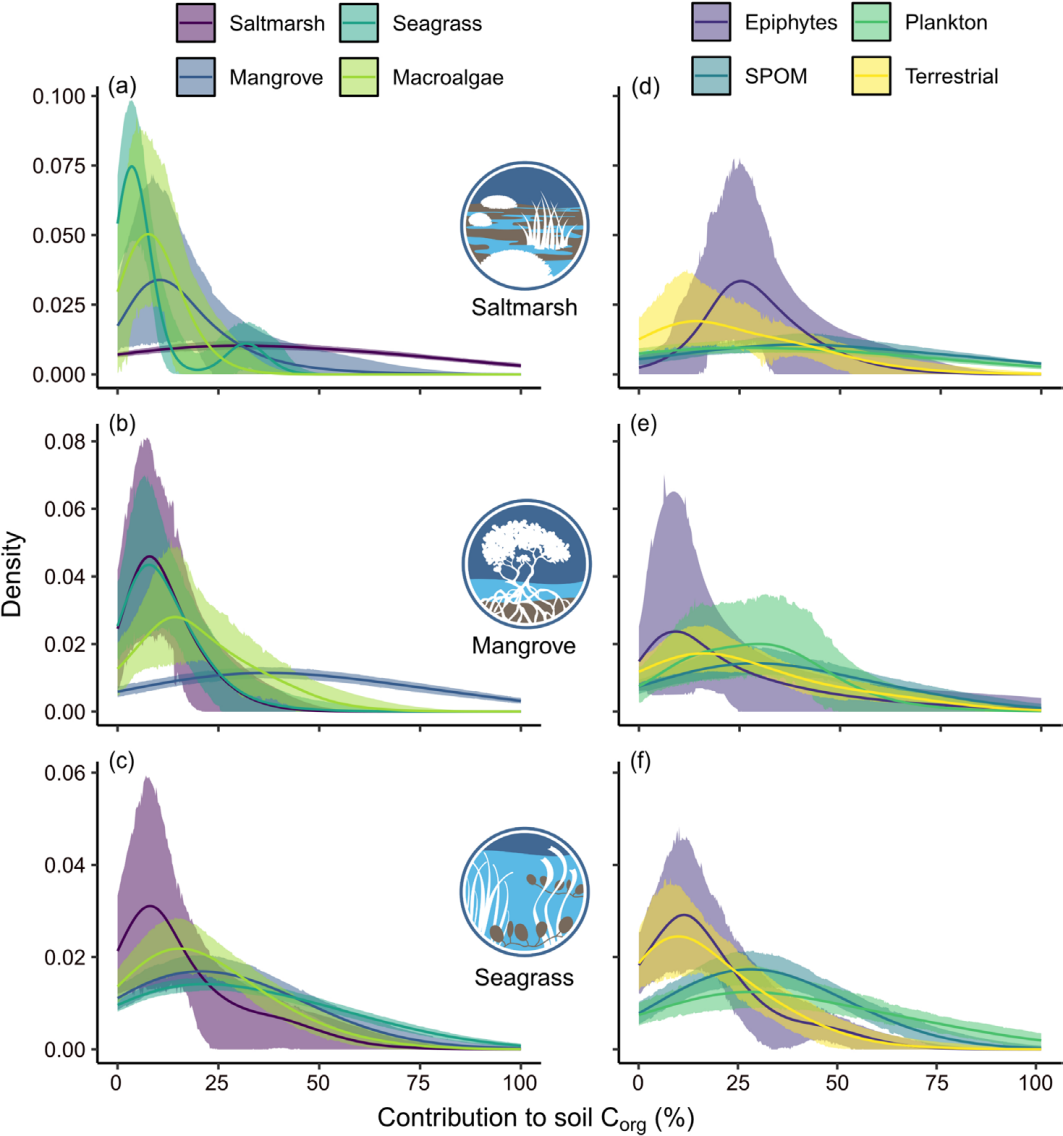
Probability density curves for the estimated contributions of different sources of organic carbon (C_org_) into soil sinks of (a, d) saltmarsh, (b, e) mangrove, and (c, f) seagrass wetlands. Summarized intervals of 1000 bootstrap simulations are based on the variance reported in each dataset. Panels on the left (a–c) indicate probable contributions of relatively discrete autotrophic groups (saltmarsh plants, mangroves, seagrass, and macroalgae) into the soil C_org_ mixture; panels on the right (d–f) are probable contributions from groups that comprise complex carbon source mixtures (epiphyte and plankton communities, suspended particulate organic matter [SPOM], terrestrial plants) that were most often explored as end‐members in the published mixing model outcomes.

Delving into the estimates for specific autotrophic sources, we find a broad portfolio of allochthonous carbon contributions to soil C_org_ at equal or greater proportions than the local autochthonous carbon sources (Figure 5, Table S2). Macroalgae, epiphytes, suspended particulate organic matter (SPOM), plankton, and terrestrial plants are potentially important C_org_ donors in all three wetland types (Figure 5). Cross‐subsidies of C_org_ flowing among wetlands from neighboring habitat‐forming vegetation are also evident, particularly seagrass C_org_ into mangrove soils (Figure 5b) and mangrove C_org_ into seagrass soils (Figure 5c and Table S1). However, these trends should be treated with some degree of caution due to the wide range of independent estimates and variances per group (Table S2).

## 4 Discussion

Autochthonous organic carbon (C_org_) fixed by habitat‐forming vegetation and buried in the soil beneath them has been a consistent focus of blue carbon research and management in coastal wetlands (Macreadie et al. 2019). Using this focus to derive an autochthonous to allochthonous carbon ratio across multiple studies, our meta‐analysis revealed a broad‐spectrum of reported contributions from seagrass, mangrove trees, and saltmarsh plants to their respective pools of soil C_org_. However, when accounting for the different study designs, we found habitat‐forming vegetation tended to contribute less than half of total soil C_org_ in all these coastal wetlands, with the specific log‐ratio of autochthonous to allochthonous C_org_ varying with wetland type, climatic zone, soil sampling design, and modeling approach. Moreover, we found considerable variation in these log‐ratios after accounting for these fixed effects, which likely arise from site‐specific conditions such as geomorphic setting, seascape composition, and local variation in the foundational wetland plants (e.g., Lavery et al. 2013; Gorham et al. 2021; Kennedy et al. 2022). Early models restricted to relatively few tracers and sources yielded higher estimates of autochthonous over allochthonous carbon contributions to wetland soils. Recognizing that seascapes are a diverse mosaic of autotrophs connected by regular water movements, studies have increasingly adopted more tracers (e.g., multiple stable isotopes, fatty acids, eDNA) and more flexible mixing models (e.g., Bayesian MixSIR, MixSIAR; Parnell et al. 2013; Stock et al. 2018) to reveal soil C_org_ in most wetlands is coming from a diversity of allochthonous sources. Based on this long history of published soil C_org_ measurements in coastal wetlands, we find a broad portfolio of photosynthetic sources collectively supporting soil carbon sequestration across connected blue carbon seascapes.

Habitat‐forming vegetation in coastal wetlands is critical to the process of carbon sequestration because it provides the physical scaffolding that stabilizes the soil and enhances the burial of suspended material arriving via tidal circulation and other hydrological features (Duarte et al. 2005; Macreadie et al. 2019). Understandably, this has focused attention on the autochthonous carbon contributions of habitat‐forming vegetation in blue carbon research, policy development, management and restoration (Howard et al. 2017; Macreadie et al. 2019, 2021). Building upon recent single‐wetland analyses (e.g., mangroves; Zhang et al. 2024), our global synthesis indicates that a mixture of other C_org_ sources can make equal or greater contributions to soil C_org_ via allochthonous pathways in three of the most prominent wetland types for blue carbon solutions to climate change. While habitat‐forming vegetation from adjacent wetlands is an important driver of that allochthonous carbon (e.g., 27% of seagrass soil C_org_ is mangrove‐derived, mean of *n* = 30 estimates; Table S1), similar contributions are evident from “emerging” blue carbon donors like macroalgae (e.g., 24% macroalgal carbon in seagrass soils, mean of *n* = 31 estimates; Table S1). Benthic macroalgae are a ubiquitous and highly productive group of photoautotrophs, but the paucity of empirical data on macroalgal carbon storage in durable sinks has hampered their inclusion in blue carbon frameworks (Howard et al. 2017; Lovelock and Duarte 2019; Macreadie et al. 2021). As these data gaps are being filled (e.g., Ortega et al. 2019; Watanabe et al. 2020; Santos et al. 2021; Pessarrodona et al. 2023), macroalgae are being incorporated into emissions frameworks (e.g., Japan's Greenhouse Gas Inventory; MoEJ 2024). For macroalgae to become a tractable component of blue carbon management and restoration, we need to adopt more specific macroalgal tracers such as genus‐level meta‐barcoding of chloroplast DNA, compound‐specific isotopes, and lipids (Kelly and Scheibling 2012; Macreadie et al. 2019; Ortega et al. 2020). This multifaceted approach will help unravel the specific actors, seasonality, and spatial pathways (e.g., epiphytic on seagrass vs. macroalgal forests; Hyndes et al. 2013; Santos et al. 2021) for macroalgal carbon sequestration across blue carbon seascapes.

Our synthesis revealed some other major blue carbon sources of interest: suspended particulate organic matter (SPOM or “seston”), plankton, and terrestrial plants. Studies that considered these sources in their mixing models estimated their relatively high contributions to soil C_org_ (habitat‐specific means of 17.2–48.8 SD%, Table S1). We see a need for caution here, because these source groups are complex mixtures that can vary considerably in chemical signature. Terrestrial plant detritus and SPOM are comprised of living and decaying organic matter of varying provenance spanning bacteria, phytoplankton, and decomposing particles of macroscopic algae and/or flowering plants (e.g., Malet et al. 2008; Liénart et al. 2017; Bruno et al. 2023). Plankton are a diverse organismal community that also vary dramatically over space and time (e.g., Colebrook 1982; Thompson et al. 2015; Holland et al. 2024). Depending on the sampling method, plankton source signatures can also be based on a mixture of autotrophs and heterotrophs (e.g., whole samples from 63 μm nets that retain both phytoplankton and zooplankton). These mixtures make it difficult to track the linkages of carbon from autotrophic fixation to soil sequestration that are necessary for net sequestration calculations and formulating blue carbon solutions (Lovelock and Duarte 2019; Macreadie et al. 2019; Howard et al. 2023). Ambiguity in these organic carbon sources also complicates the interpretation of mixing model outcomes. For example, when SPOM is a mixture of organic matter from mangroves, seagrass, and macroalgae, the SPOM tracer signature will be intermediate to these distinct carbon sources and will often most closely match the soil signatures. Consequently, a typical mixing model may allocate a higher contribution from SPOM to the soil mixture. How should this result be interpreted in the context of understanding and managing a blue carbon seascape? The original source of carbon is not SPOM; rather, it is the photoautotrophs that fixed the organic carbon before it entered the SPOM carbon pool. We suggest that if groups like plankton and SPOM are included as end‐members, there should be an attempt to unravel the original C_org_ sources in these mixtures (e.g., using a combination of chlorophyll *a* and N:C ratios, Watanabe et al. 2019) to allow linkage between the mixing model outcomes and the specific autotrophs that underpin blue carbon drawdown and sequestration.

The efficacy of carbon mixing models is contingent on the use of tracers with minimal signature overlap among the potential source groups (Parnell et al. 2013; Nielsen et al. 2018; Stock et al. 2018). In practice, this requires a priori decisions on obtaining enough samples for a representative tracer signature from each source, recognizing the uncertainty in those estimates, and deciding on acceptable overlap for mixing model applications. Using diagnostic approaches like iso‐space plots can help investigators identify the potential for missing end‐members and whether there are systematic shifts in tracer signature from sources to soil sink from fractionation effects (Moore and Semmens 2008; Parnell et al. 2013). Although fractionation in stable isotope signatures among consumers and their prey has become regularly assessed in trophic studies (typically called “discrimination factors”; Nielsen et al. 2018; Stephens et al. 2023), such effects are rarely considered in soil carbon research (Macreadie et al. 2019; Ward et al. 2024). This is despite the fact stable isotope signatures can vary strongly among living tissues on the same plant type (e.g., 3.2‰ and 1.8‰ in δ^13^C among leaves and roots of mangroves and seagrasses, respectively; Vonk et al. 2008; Kelleway et al. 2018). Moreover, signatures can also differ among the same plant types along environmental gradients (e.g., 5.4‰ δ13C among seagrasses spanning 5–35 m water depth; Cooper and DeNiro 1989) and bioregions (Ward et al. 2024). By carefully considering the environmental context and what tissue types may be most relevant to the soil carbon burial of interest (e.g., allochthonous lateral carbon flows of mangrove leaf litter and buoyant fruits), we recommend investigators apply tissue‐specific source signatures to reduce uncertainty in mixing model outcomes. Fractionation effects can also arise from bacterial decomposition during the transport of organic carbon from living source to soil sink (Smith 1981), which will vary among different types of chemical tracer and be particularly acute for some rapidly oxidized and/or metabolized compounds (e.g., fatty acids). These rates of change from decomposition remain poorly understood, particularly how the degradability of organic carbon (the primary element of interest) differs from other potential tracers (e.g., lipids, eDNA) of organic carbon (Macreadie et al. 2019). However, data on shifts in the isotopic signature of blue carbon sources during degradation are emerging (Kelleway et al. 2022), and these should be used to make appropriate adjustments to carbon isotope signatures before undertaking mixing model calculations (Nielsen et al. 2018; Stock et al. 2018).

Modifications in tracer signatures within the soil profile are also relevant to understand the differences in C_org_ composition that were highlighted in our meta‐regression. Changes in both C_org_ concentrations (general decline with soil depth) and tracer signatures that occur over time are captured down the soil profile. These variations with soil depth can arise due to bacterial decomposition, chemical diagenesis, climatic effects, changes in vegetation type, and coastline geological evolution (Krüger et al. 2024). As a consequence, contemporary source signatures obtained from living tissues may not provide an effective match to historical organic carbon buried in the soil decades to centuries ago. One remedy is using soil dating techniques (e.g., ^210^Pb or ^14^C dating; Canuel and Hardinson 2016; Arias‐Ortiz et al. 2018) to make adjustments for known shifts in stable isotope enrichment through time. For example, adjusting for the 3.8‰ shift in δ^13^C of C3 plant tissues caused by the increased burning of fossil fuels since 1850 (Krüger et al. 2024). Major shifts in wetland habitat type over epochs of sea‐level change and coastal land‐use changes may also be captured in long soil cores (Hagger et al. 2022; Murray et al. 2022; Kirwan et al. 2023). Disentangling these drivers of varying C_org_ signatures with soil depth will require careful application of high‐preservation coring techniques and soil dating metrics appropriate to the timescales of interest (Kelleway et al. 2017; Arias‐Ortiz et al. 2018). Since historical tracer signatures are unlikely to be obtained for different organic carbon sources, sensitivity analyses may provide a tractable means to explore the potential for changes in vegetation type to have caused soil depth modifications in C_org_ signatures over time (e.g., Drexler et al. 2020).

Embracing a wide diversity of potential C_org_ sources into blue carbon mixing models will require further innovation, and we can look to trophic applications of stable isotope mixing models for some signposts (Nielsen et al. 2018; Geraldi et al. 2019; Stephens et al. 2023). A key issue is overcoming extensive overlap in tracer signatures among sources, which is particularly acute for the commonly used bulk tracer δ^13^C (Geraldi et al. 2019). In studies of complex food webs, the solution has been to include additional tracers, such as stable isotopes of other elements, compound‐specific stable isotopes, and/or fatty acids (Geraldi et al. 2019; Pickett et al. 2024). Indeed, fatty acids have been found to be honest tracers of macroalgal carbon in experimental soil mixtures (Erlania et al. 2024). However, some of these tracers can yield a high number of variables (e.g., > 50 fatty acids per source), which can be autocorrelated or indistinct among carbon sources, so we recommend selecting a subset of tracers via targeted source compositions (e.g., selection of fatty acids specific to algae; Pickett et al. 2024) or statistical approaches (e.g., constrained ordination, Neubauer and Jensen 2015; similarity matrix weightings, Pickett et al. 2024). Bayesian mixing models have been developed to utilize multiple tracer types, either to derive joint estimates (e.g., stable isotopes and fatty acids together; Neubauer and Jensen 2015; Brett et al. 2016; O'Donovan et al. 2018) or via application of informative priors that inform the mixing model based on a different tracer type (Chiaradia et al. 2014; McInerney et al. 2020; Pickett et al. 2024). Given the wide number of C_org_ source groups driving a portfolio of blue carbon sequestration in diverse and connected seascapes, this application of multiple lines of evidence will need to become standard for future blue carbon work.

Resilient coastal wetlands that continue to deliver a nature‐based solution to carbon sequestration under rapid climate change will require a flexible blue carbon seascape model that underpins management, conservation, and restoration actions. Our meta‐analysis highlights the breadth of plant and algal sources that support soil carbon sequestration in tropical and temperate wetlands. The relative importance of these sources varied considerably among studies, which to some extent was due to study design and modeling approach, but site‐specific conditions will also be significant. For instance, large‐scale climatic zone effects on wetland species composition are known to interact with local geomorphic and hydrodynamic settings to shape the specific sources, pathways, and rates of C_org_ accumulation in coastal wetlands (e.g., Lavery et al. 2013; Gorham et al. 2021; Kennedy et al. 2022). In that context, we need more spatially explicit analyses to reveal the relative predictive power of these and other spatial context effects, such as the surrounding landform and areal extent of neighboring plant carbon sources (e.g., Quak et al. 2016; Asplund et al. 2021; Maxwell et al. 2024). By including source habitat area and other spatial covariates within flexible mixing analyses that embrace a wide diversity of carbon sources, we can build the spatial models of blue carbon flows needed to understand the consequences of varying seascape configurations. When combined with an understanding of how chronic and acute disturbances are likely to reconfigure our coastal seascapes (a priority outlined in Macreadie et al. 2019), we can take the adaptive management actions necessary to support a resilient portfolio of soil carbon sequestration in blue carbon wetlands of the Anthropocene.

## Author Contributions

**Christopher J. Fulton:** conceptualization, data curation, funding acquisition, investigation, methodology, project administration, writing – original draft, writing – review and editing. **Diego R. Barneche:** conceptualization, data curation, formal analysis, investigation, methodology, software, visualization, writing – review and editing. **Kay Davis:** data curation, investigation, methodology, writing – review and editing. **Cal Faubel:** data curation, investigation, methodology, visualization, writing – review and editing. **Cecilia Pascelli:** data curation, investigation, methodology, writing – review and editing. **Julie Vercelloni:** data curation, formal analysis, investigation, methodology, software, validation, writing – review and editing. **Shaun K. Wilson:** conceptualization, data curation, investigation, methodology, writing – review and editing.

## Conflicts of Interest

The authors declare no conflicts of interest.

## Supporting information


**Data S1:** gcb70420‐sup‐0001‐Supinfo.pdf.

## Data Availability

The data that support the findings of this study are openly available in the Australian Institute of Marine Science Data Centre at https://doi.org/10.25845/jt3q‐4y65.
