# Supplementary material for: Tracing Blue Carbon Flows Across Diverse Seascapes
Source: Glob Chang Biol. 2025 Aug 15;31(8):e70420. doi: 10.1111/gcb.70420 (PMC12355348; doi:10.1111/gcb.70420)
Supplement: Supplementary file 1 — Data S1: gcb70420‐sup‐0001‐Supinfo.pdf. [file GCB-31-e70420-s001.pdf]

Supplementary Materials

Tracing blue carbon flows across diverse seascapes

Christopher J. Fulton*[1,2], Diego R. Barneche[1,2], Kay Davis[1], Cal Faubel[1], Cecilia Pascelli[1], Julie Vercelloni[3,4], Shaun K. Wilson[1,2]

[1]Australian Institute of Marine Science, Crawley, Western Australia 6009

[2]Oceans Institute, University of Western Australia, Crawley, Western Australia 6009

[3]Australian Institute of Marine Science, Cape Cleveland, Queensland 4810

[4]Centre for Data Science, Queensland University of Technology, Brisbane, Queensland 4000

*Corresponding author: chris.fulton.au@gmail.com

Standardised variance terms

Not all studies (18 of 110, i.e., 16.4%) reported the standard deviation (SD) as a measure of spread around the estimated autochthonous organic carbon contribution to soil samples (%). For all these cases, we approximated the reported metric (α) to SD (hereafter, adjusted SDs) by assuming a normal distribution, as follows: SD=α×√(N)/1.96 for studies reporting 95% credible intervals (we used half of the value, so 47.5% C.I.), which were all based on Bayesian models (n = 13); SD=α/6 for studies which only reported the full range of values (n = 2), assuming the range to cover 6 standard deviations (i.e., % of the distribution; SD=α√N) for studies reporting the standard error of the mean (n = 2); and SD=α×1.4826 for studies reporting the median absolute deviation (MAD; n = 1).

Leave-one-out cross-validation

We acknowledge that our list of predictors was in part decided by information that was consistently reported across the 100 studies in our compilation. It is possible that more, or none of the predictors might have a predictive effect on our response (i.e., auto-to-allo log-ratio). Although we cannot investigate the former case due to obvious lack of data, we did verify whether our full model had higher predictive performance than a null intercept-only meta-regression model, without any "fixed" effect covariates. We compared both models using leave-one-out cross-validation (LOO), which, similarly to widely applicable information criterion (WAIC), is a fully Bayesian model selection procedure for estimating pointwise out-of-sample prediction accuracy (Hooten & Hobbs 2015; Vehtari et al. 2017). For each model, we calculated the expected log pointwise predictive density (ELPD) using the log-likelihood evaluated at the posterior simulations of the parameter values (Vehtari et al. 2024). The pairwise differences between both models, ELPD (full - null) was -10.8 +/- 5.7 (standard error), indicating that the full model has substantial predictive capacity over the null model.

Posterior predictive checks

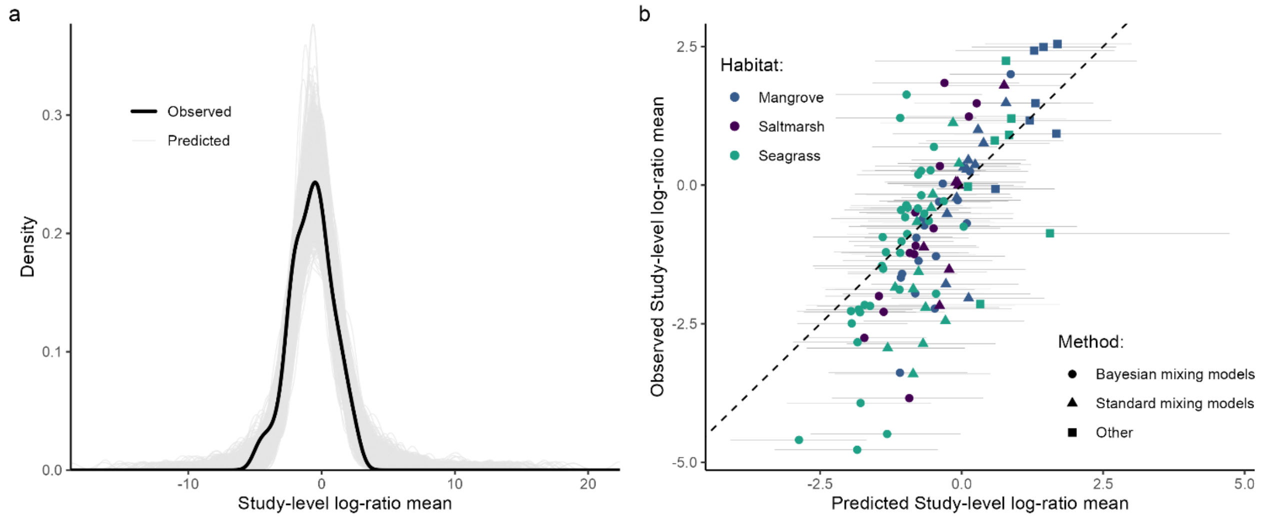

Figure S1. Posterior predictive checks: (a) density overlay curves depicting how 1,000 randomly sampled posterior predictions (thin grey lines) compare against the observed distribution of log-ratios; and (b) predicted (mean and 95% uncertainty intervals represented by horizontal lines) vs. observed, with the dashed line representing a 1-to-1 relationship.

Residual diagnostics

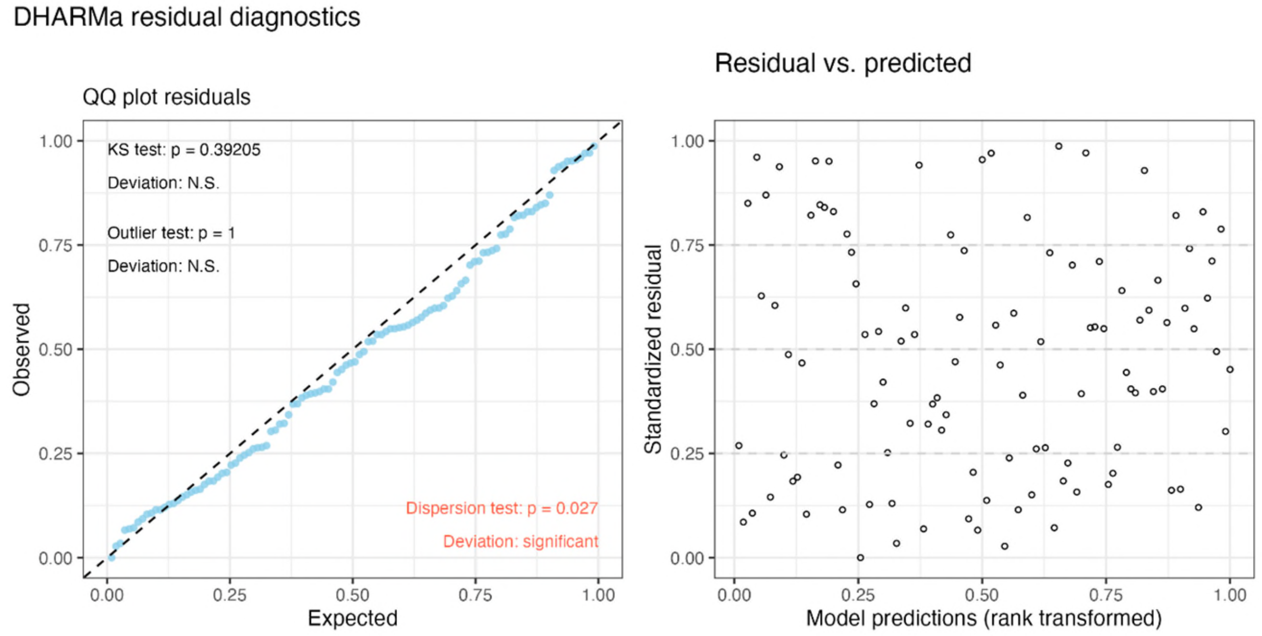

Figure S2. Residual diagnostics using DHARMa (Hartig 2022): (left panel) QQ-plot to detect overall deviations from the expected distribution, by default with added tests for correct distribution (KS test), dispersion and outliers; and (right panel) residuals against the predicted value. For further details see Hartig (2022).

Meta-regression model summary

Table S1. *Full meta-regression model summary with reference levels for the intercept estimate of the log-ratio of autochthonous to allochthonous carbon in tropical mangrove soil estimated via Bayesian mixing models, and continuous covariates (soil sampling depth, number of end members considered, and number of replicate soil samples) scaled to zero. Interpreting predictions of log-ratios in this model, and therefore of the relative contributions of autochthonous and allochthonous carbon, depends on the conditioning statements from all covariates.*

**Coefficient**	**Estimate** (lower and upper 95% Bayesian uncertainty intervals)	$\hat{R}$	**Effective Sample Size**
Intercept	(-0.94) **-0.47** (-0.01)	1.00	9,607
Temperate	(-0.24) **0.28** (0.80)	1.00	11,222
Saltmarsh	(-1.15) **-0.43** (0.29)	1.00	10,320
Seagrass	(-1.42) **-0.89** (-0.36)	1.00	10,144
Standard mixing models	(0.00) **0.54** (1.06)	1.00	14,163
Other analysis types	(1.01) **1.71** (2.40)	1.00	12,921
Soil depth (scaled)	(-0.37) **-0.13** (0.11)	1.00	13,859
No. end-members (scaled)	(-0.43) **-0.03** (0.37)	1.00	17,128
No. soil replicates (scaled)	(-0.05) **0.27** (-0.59)	1.00	14,850

Reported contributions from specific organic carbon sources

Table S2. *Mean ± Standard Deviation (number of datasets) of estimated organic carbon (C_{org}) contributions (% of total soil organic carbon) from different photo-autotrophic sources (first column) into the soil of three sink habitats (columns 2-4) from 78 published studies. Bold values represent the autochthonous carbon pathway; all others are potential allochthonous contributions. Totals do not sum to 100% per sink habitat (column) because not all studies considered the same spread of source categories in their mixing models. Values are means and variance for only the population of datasets (n indicated in parentheses) that considered that source in their mixing model outcomes.*

**C_{org} source**	**Sink habitat**		
	**Saltmarsh**	**Mangrove**	**Seagrass**
Saltmarsh	**41.0 ± 25.4 (23)**	10.6 ± 4.9 (6)	17.3 ± 14.1 (9)
Mangrove	17.4 ± 10.7 (3)	**49.5 ± 24.3 (35)**	26.9 ± 19.4 (30)
Seagrass	7.3 ± 10.3 (8)	10.1 ± 5.8 (14)	**32.9 ± 22.4 (59)**
Macroalgae	7.5 ± 5.9 (3)	20.3 ± 10.2 (8)	24.1 ± 14.1 (31)
Epiphytes	35.6 ± 18.6 (2)	21.0 ± 22.6 (2)	16.6 ± 13.7 (10)
Plankton	34.8 ± 27.4 (6)	26.9 ± 15.7 (5)	44.1 ± 28.3 (13)
SPOM	48.8 ± 24.9 (8)	38.6 ± 20.8 (17)	37.0 ± 19.8 (22)
Terrestrial	38.9 ± 20.8 (10)	26.8 ± 20.7 (13)	17.2 ± 14.4 (21)

References

Hartig F (2022) DHARMa: residual diagnostics for hierarchical (multi-level/mixed) regression models. https://cran.r-project.org/web/packages/DHARMa/vignettes/DHARMa.html

Hooten MB, Hobbs NT (2015) A guide to Bayesian model selection for ecologists. *Ecological Monographs* 85, 3–28. doi: 10.1890/14-0661.1.

Vehtari A, Gelman A, Gabry J (2017) Practical Bayesian model evaluation using leave-one-out cross-validation and WAIC. *Statistics and Computing* 27, 1413–32. doi: 10.1007/s11222-016-9696-4.

Vehtari A, Gabry J, Magnusson M, Yao Y, Bürkner P, Paananen T, Gelman A (2024). loo: Efficient leave-one-out cross-validation and WAIC for Bayesian models. R package version 2.8.0 https://mc-stan.org/loo/
